# Nuss procedure for combined pectus excavatum and carinatum in a patient with a history of congenital esophageal atresia repair surgery

**DOI:** 10.1186/s13019-022-01759-0

**Published:** 2022-01-15

**Authors:** Gyeol Yoo, Jin Yong Jeong

**Affiliations:** 1 Department of Plastic and Reconstructive Surgery, Incheon St. Mary’s Hospital, College of Medicine, The Catholic University of Korea, Seoul, Republic of Korea; 2 Department of Thoracic and Cardiovascular Surgery, Incheon St. Mary’s Hospital, College of Medicine, The Catholic University of Korea, Seoul, Republic of Korea; 3 Department of Thoracic and Cardiovascular Surgery, Incheon St. Mary’s Hospital, College of Medicine, The Catholic University of Korea, 56 Dongsu-ro, Bupyeong-gu, Incheon, 21431 Republic of Korea

**Keywords:** Pectus excavatum, Pectus carinatum, Nuss procedure, Cardiothoracic surgery, Congenital esophageal atresia

## Abstract

Cardiothoracic surgery usually causes tissue adhesion on the operation site which increases the risk of complications in the subsequent thoracic surgery including Nuss procedure. Disorders that require cardiothoracic surgery include chest wall deformities such as pectus excavatum, congenital heart diseases, lung diseases such as congenital cystic adenomatiod malformation and bronchopulmonary dysplasia, and congenital diaphragmatic hernia. Recently, we encountered a rare case of combined pectus excavatum and carinatum in a patient with a history of congenital esophageal atresia repair surgery. Commendably, despite tissue adhesion from the previous surgery, a modified Nuss procedure was performed successfully with no complications. We agree that the Nuss procedure is feasible for thoracic deformities in patients with a surgical history of cardiothoracic surgery.

**Correspondence**

**Dear Sir,**

Our study was inspired by Takanari et al. who presented six cases of the Nuss procedure for pectus excavatum in patients with a history of intrathoracic surgery [1]; four cases of congenital cystic adenomatoid malformation and two cases of congenital diaphragmatic hernia. Cardiothoracic surgery usually causes pleural adhesions and significant adhesiolysis may be required during a subsequent thoracic surgery [2]. They performed ultrasonography examination to identify the pleural adhesions before the operation. With the assistance of thoracoscopy they dissected the pleural adhesions with Maryland bipolar forceps and freed the lung visceral pleura from the thoracic wall before performing the Nuss procedure. Recently, we encountered a rare case of combined pectus excavatum and carinatum with a history of congenital esophageal atresia repair surgery.

A five-year-old male presented with anterior chest wall deformity and chest discomfort. He had a history of thoracotomy and laparotomy for congenital esophageal atresia at one month of age. A chest computed tomography (CT) revealed a combined pectus excavatum and carinatum with mild depression of the right anterior chest wall and protrusion of the sternal portion. A gastric conduit formed after esophageal resection was also observed in the right posterior mediastinum (Fig. 1A). The patient underwent a needlescope-assisted modified Nuss procedure for correction of pectus excavatum and carinatum. A 2-mm needlescope with carbon dioxide insufflation showed localized pleural adhesions along the previous thoracotomy site, depressed right costal cartilages, and stomach (Fig. 1C, D). After performing adhesiotomy with a 2-mm endo-scissors inserted through the additional anterior chest wall port, two metal bars were inserted in a cross form to elevate the depressed chest wall. Then a Number 5 Ethibond Excel (Ethicon, Somerville, NJ) was inserted in the sternal subcutaneous tissue to compress the protruding sternal area. Hemovac drains remained in both pleural cavities. On the 1st and 4th postoperative days, the left and right drains were removed sequentially. Postoperative CT confirmed that the Nuss procedure showed that the anterior chest wall deformities were successfully corrected (Fig. 1B). The patient was followed up for six months with no surgical complications.

**Fig. 1:**
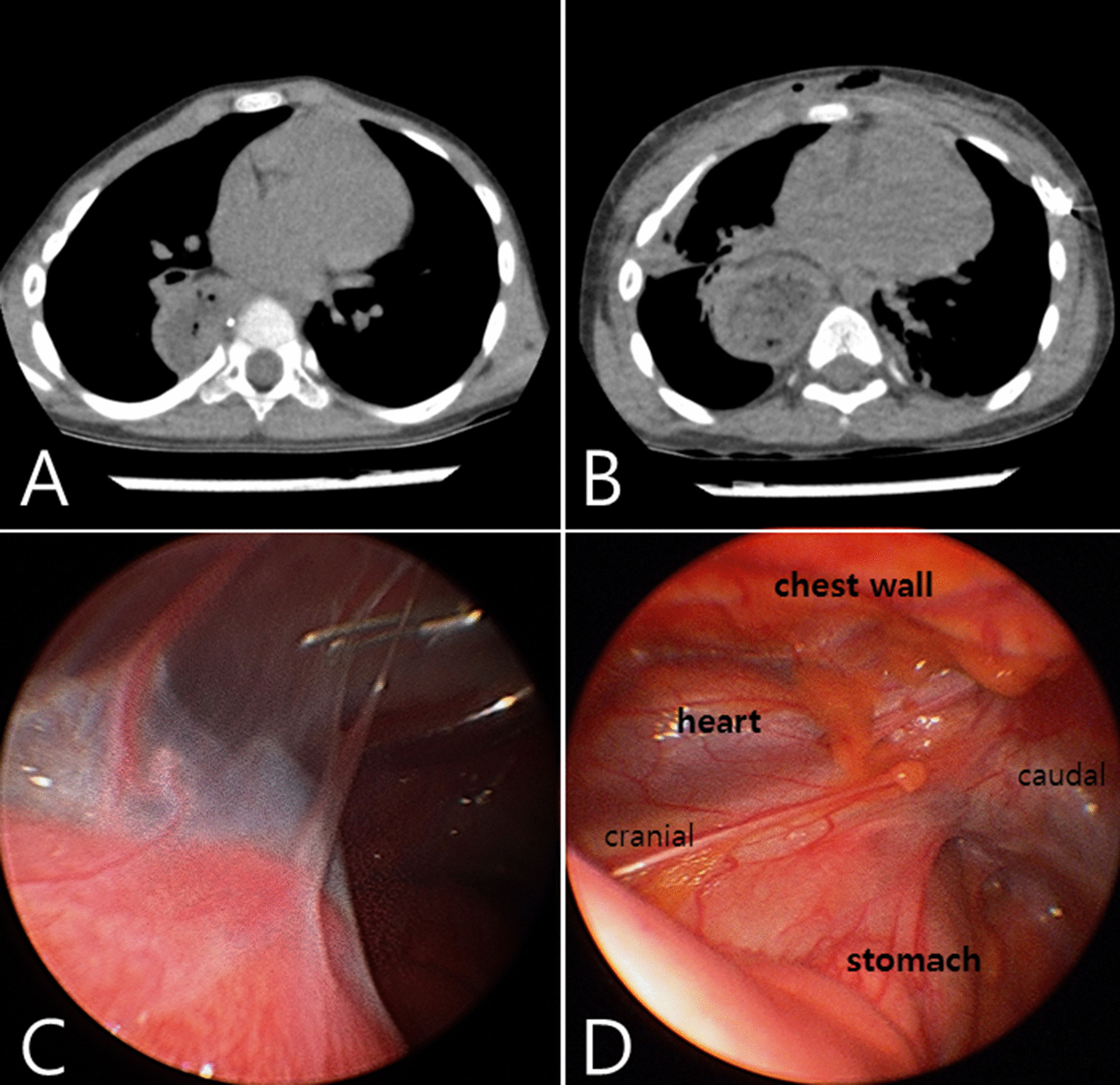
**A** Preoperative computed tomography (CT) showing depression of the right anterior chest wall with sternal protrusion and previously operated stomach in right side mediastinum. **B** Postoperative CT after insertion of two pectus bars and polyester suture. **C** A 2-mm needlescope showing localized pleural adhesions along the previous thoracotomy site. **D** Depressed right costal cartilages and stomach

Nuss procedure in patients with a history of cardiothoracic surgery is associated with a high rate of complication. Guo et al. reported that the complication rate was 21.4% for secondary Nuss procedure patients and 13.8% for primary Nuss procedure patients [3]. Interestingly, our study presents an exceptional case of a successful Nuss procedure that was performed without complications secondary to cardiothoracic surgery for congenital esophageal atresia repair.

Redlinger et al. performed Nuss procedures in 100 patients with recurrent pectus excavatum [2]. Of them, 42 patients have undergone Ravitch procedure and 51 patients Nuss procedure. The bar displacements rate after the Nuss procedure was 12% in the Ravitch group and 7.8% in the Nuss group. Although they were not fatal complications, two major intraoperative cardiac arrests occurred due to an acquired thoracic chondrodystrophy in the Ravitch group. They found that extensive pleural adhesion necessitating decortication can occur after Nuss and Ravitch procedures and reported more than 95% success rate of the subsequent Nuss procedure regardless of initial repair technique. Li et al. performed a Nuss procedure in 30 patients with pectus excavatum which subsequently developed after congenital heart disease surgery [4]. Postoperative complications rate was 16.7%; the complications included pneumothorax, wound hematoma, pericardial penetration, and bar displacement. Although substernal adhesions, which increase the risk of cardiac injury, were present in all the patients, most of them were successfully dissected and 96.7% excellent Nuss procedure results were reported. Takanari et al. performed Nuss procedure in 6 patients with a history of intrathoracic surgery for congenital cystic adenomatoid malformation repair and congenital diaphragmatic hernia repair [1]. Postoperative complications occurred in one patient (17%). Pleural adhesions were observed in all the patients and dissection was done uneventfully with the assistance of ultrasound, thoracoscope, and bipolar forceps. These findings demonstrate the efficacy of Nuss procedure correcting pectus excavatum in patients with a history of a previous pectus excavatum surgery, congenital heart surgery, and pulmonary and diaphragmatic surgeries. Similarly, our case was successful with excellent results and no surgical complications.

In summary, we encountered a rare case of combined pectus excavatum and carinatum with a history of congenital esophageal atresia repair surgery. In spite of tissue adhesion following the previous surgery, a modified Nuss procedure was successful with no complications. Our findings are in agreement with those of Takanari’s that demonstrated the Nuss procedure as an effective and safe approach for thoracic deformities in patients with a history of cardiothoracic surgery.

## Data Availability

Not applicable.

## Abbreviation

CT: Computed tomography
